# The importance of inflammatory biomarkers in detecting and managing latent tuberculosis infection

**DOI:** 10.3389/fimmu.2025.1538127

**Published:** 2025-02-06

**Authors:** Harinisri Gunasekaran, Uma Devi Ranganathan, Ramalingam Bethunaickan

**Affiliations:** ^1^ Department of Immunology, ICMR-National Institute for Research in Tuberculosis, Chennai, India; ^2^ University of Madras, Chennai, India; ^3^ Faculty of Medical Research, Academy of Scientific and Innovative Research (AcSIR), Ghaziabad, India

**Keywords:** tuberculosis, biomarker, latent TB, inflammation, cytokine, chemokine, acute phase proteins

## Abstract

Infection with *Mycobacterium tuberculosis* (Mtb) triggers an autoimmune-like response in the host leading to further complications. One of the major concerns in eliminating Tuberculosis (TB) is identifying individuals with Latent Tuberculosis Infection (LTBI) who serve as major reservoirs of Mtb making them the important target group for TB eradication. Since no gold standard tests are available for detecting LTBI, the global burden of LTBI cannot be precisely determined. Since LTBI poses several challenges to worldwide healthcare, managing LTBI must be the key priority to achieve a TB-free status. The inflammatory mediators play a major role in determining the outcome of the Mtb infection and also their levels seem to change according to the disease severity. Identification of inflammatory mediators and utilizing them as diagnostic biomarkers for detecting the various stages of TB disease might help identify the reservoirs of Mtb infection even before they become symptomatic so that preventative treatment can be started early. In summary, this review primarily focuses on exploring different inflammatory markers along the course of the Mtb infection. Identifying LTBI-specific biomarkers helps to identify individuals who are at higher risk of developing TB and preparing them to adhere to preventive therapy thus minimizing the global burden of TB.

## 1 Introduction

LTBI is a stage of consistent immunological response to stimulation by Mtb antigens but without any clinical and radiological manifestations of active TB disease (1). Mycobacteria is capable of triggering an autoimmune-like response in the host. Almost 33% of the global population, approximately 2.3 billion people, have LTBI (2). LTBI is manifested as an asymptomatic infection and the majority of them never develop TB disease in their whole lifetime. But in the minority of those people with weak immune system, the bacteria becomes active and progresses to TB disease (3). Reactivation of LTBI to transmissible disease can happen even decades after first exposure. Therefore, the high incidence of LTBI is considered a major obstacle to the worldwide eradication of TB (4). Thus, one of the priority areas of the World Health Organization’s (WHO) End TB Strategy is the detection, treatment and management of LTBI (5). Therefore, the primary aim of this review is to address the inflammatory process involved in LTBI and the utility of new immune-based diagnostic biomarkers to develop better tools to address TB control.

## 2 Screening and diagnosis of LTBI

Diagnosing LTBI at an early stage is an important step to take preventative action against the development of active TB. LTBI is characterized by its asymptomatic state and undefined pathophysiology. It is identified only through immunological evidence obtained by stimulating with Mtb antigens although this cannot be equivalent to the presence of live Mtb in the host. There is no gold standard test to detect LTBI. However, there are two principle immune-based diagnostic tests currently used in clinical research to identify latent infection: *in-vivo* tuberculin skin test (TST) and ex-vivo interferon-gamma release assay (IGRA) in addition to chest radiography, physical examination, prior exposure, and medical history (6, 7). TST is simple to perform and inexpensive, but it has poor specificity partly because of false positives associated with the Bacillus Calmette-Guerin (BCG) immunization. It uses an antigenic mixture extracted as a purified protein derivative (PPD) from the supernatant of Mtb liquid cultures (7, 8). It was the only test used to diagnose LTBI until 2001. In 2001, the commercially available IGRA kits, the QuantiFERON-TB test (QFT) and T-SPOT.TB were approved as an aid for detecting LTBI. They are *in-vitro* diagnostic kits that quantify the interferon-gamma (IFN-γ) secreted by lymphocytes incubated with TB-specific antigens. IGRA results are less susceptible to reader bias and error than TST. IGRA is quite expensive and shows higher sensitivity in BCG-vaccinated individuals. Because of its better specificity than TST, IGRA is utilized in routine LTBI screening for preventive treatment. However, IGRA is similar to TST in terms of active TB detection (7, 9).

Both these existing diagnostic tools can detect immune responses to Mtb but cannot distinguish between latent infection and active TB. Thus, due to the low predictive value of these biomarkers, new biomarkers are needed to address this limitation. Interestingly, inflammatory biomarkers, can better reflect the ongoing inflammatory process associated with active TB. Therefore, these limitations can be overcome by the incorporation of inflammatory biomarkers into diagnostic algorithms which can significantly improve these processes by providing a quantitative layer to the assessment of TB disease, resulting in more accurate and fast diagnoses. Inflammatory biomarkers can provide additional diagnostic value by reflecting the ongoing inflammatory process. An increased levels of pro-inflammatory cytokines are commonly seen in active TB due to the ongoing infection and inflammation, but they are often normal or only slightly elevated in LTBI. They are also less expensive and more accessible diagnostic option.

## 3 Stages of LTBI

Classically, Mtb infection was thought to manifest in humans either as a latent infection or an active disease. But in fact, Mtb infection results in a wide range of clinical manifestations in humans. Although LTBI is commonly associated with the containment of bacteria in an inactive form, the current classification will encompass a wide variety of intermediate states, from those who have fully recovered from the infection to those who are incubating actively replicating bacteria without any clinical symptoms (10). Just as active TB has different severity levels, there exists a wide spectrum of outcomes in latent infection. The outcome of the infection is determined by the bacteria’s genetic and phenotypic variety as well as the way these bacteria interact with the hosts. Differentiating between different disease stages play a critical role in patient care and public health as it ensures appropriate treatment, prevents transmission, aids in the management of drug resistance, effective monitoring of progression or remission, and prevents resource wastage.

The latent infection is identified as having a normal chest radiograph without the clinical signs and symptoms of active disease and it is diagnosed exclusively by immunologic tests (TST or IGRA) (11). LTBI is a state in which the host can manage the infection but cannot get rid of all the bacteria making latently infected individuals the biggest pool of possible carriers for potential TB transmission. Reactivation, the resurgence of TB after another infection, poses the highest risk to people with latent infection (12). The latency is the most common course of infection whereas it may also rapidly or slowly progress to active TB or it may undergo a period of cycling through incipient and subclinical TB before becoming symptomatic disease. The manifestations of the disease and the duration of each stage are determined by the host’s immune response. However, spontaneous recovery may also transpire in any of these clinical trajectories.

The incipient state refers to an early, confined form of the disease that is usually asymptomatic without any radiological or microbiological evidence of active TB disease but is likely to progress to active TB without treatment.

In contrast, subclinical TB is a stage in which the host harbors viable Mtb bacteria without any clinical symptoms but can be detected through microbiologic or radiologic techniques (13, 14). In particular, people living with subclinical TB are threatening to the community as they may not exhibit any symptoms but they can still harbor the bacteria and transmit it to others. Because these subclinical cases frequently have a slower course, they might linger in the population for longer durations of time without being diagnosed, giving extended opportunities for transmission (15).

The idea of this concept of LTBI spectrum broadens the definition of TB beyond a binary status. Understating from this perspective helps us to prioritize preventive treatment, better differentiate an individual’s risk of LTBI reactivation and highlight the variability of host responses to Mtb infection. The stages of TB disease spectrum has been summarized in **Table 1**.

**Table 1:** A broader overview of the spectrum of TB disease.

TB DISEASE SPECTRUM	SELF- CLEARED	LTBI	INCIPIENT TB	SUBCLINICAL TB	ACTIVE TB
Symptoms	Asymptomatic	Asymptomatic	Asymptomatic	Asymptomatic or mildly symptomatic	Symptomatic
IGRA status	Negative	Positive	Positive	Positive	Positive
Bacillary burden	Nil	Low	Low	Moderate	High
Chest X ray	No abnormalities	No abnormalities	No/Minimal abnormalities	Minimal abnormalities	Extensive abnormalities
Sputum smear	Negative	Negative	Negative	Negative/Positive	Positive
Gen Xpert PCR	Negative	Negative	Negative	Negative/Positive	Positive
Infectiousness	No	No	No	May or may not be infectious	Yes
Treatment	None	None/Preventive therapy	None/Preventive therapy	Preventive therapy	Anti-tuberculosis therapy (ATT)

## 4 Pathogenesis of Mtb

**Figure 1:**
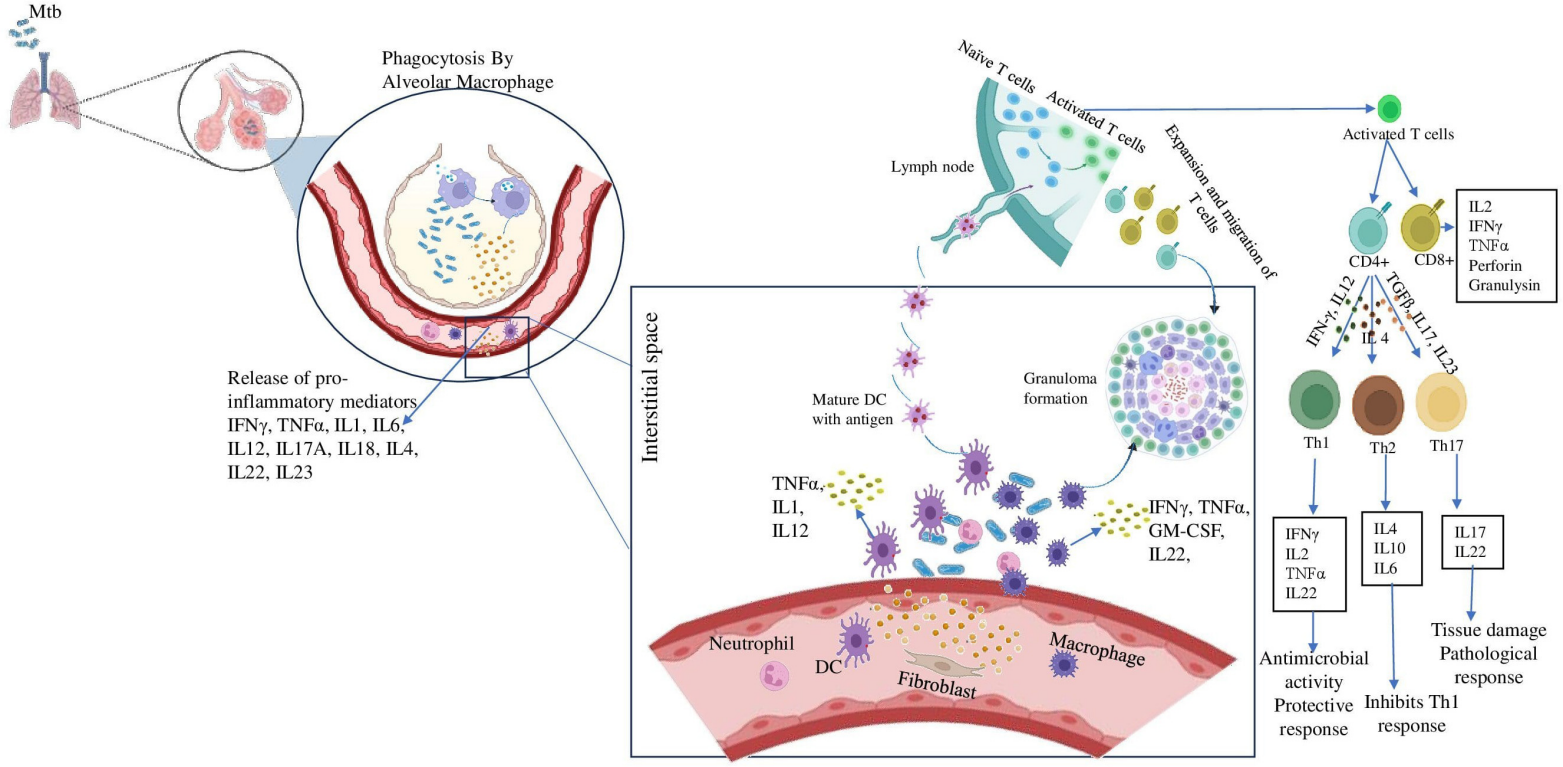
Role of inflammatory cytokines in establishing granuloma formation. After inhalation of Mtb containing aerosols, the alveolar macrophages come to the first line of defense. They phagocytose the pathogen and release pro-inflammatory cytokines like IFN-γ, TNF-α etc. These further recruit immune cells like DCs, neutrophil, fibroblast to the site of infection and they release a milieu of cytokines. Meanwhile, DCs carrying the antigen reaches the lymph node and present it to the naïve T cells and activates them. The activates T cells differentiate into CD4+ and CD8+ cells. The CD4 + cells further may differentiate into either Th1 cells in presence of IFN-γ, IL12 or Th2 cells in presence of IL4 or Th17 cells in presence of TGFβ, IL17, IL23. These cells also reach the site of infection and assemble around the bacteria to form the granuloma.

TB is an airborne disease caused by inhaling aerosolized droplet nuclei containing Mtb generated by patients with active TB. The risk of developing infection is determined by various factors such as the source, the bacilli load and the immunity power of the host (16). The bacilli that enter the alveoli are engulfed by phagocytic immune cells such as alveolar macrophages and dendritic cells. Mtb undergoes intracellular replication which then reaches the bloodstream by being carried across the alveolar wall by the immune cells (17). The bacilli-carrying dendritic cells reach the mediastinal lymph nodes where the adaptive immune response is initiated which stops further replication of the bacteria. Adaptive immune response generates granuloma which is illustrated in **Figure 1** is a collection of inflammatory cells that form a physical barrier limiting the further dissemination of the bacteria (10). The adaptive immune response is essential for both the effective control of bacterial replication and for developing protective immunity against Mtb. This is best illustrated by the extreme vulnerability of lymphopenic HIV patients to Mtb infection (18).

### 4.1 Innate immune response against Mtb

As soon as the Mtb enters the host, the host immune system recruits the major innate immune cells such as alveolar macrophages, dendritic cells (DCs), natural killer (NK) cells and neutrophils to the site of infection. These early interactions between Mtb and the host play an important role in the establishment of the infection and dictating the clinical outcome (19). Our knowledge of the initial stages of Mtb infection is quite lacking. This results from the challenge of determining when an infection first appears, for which appropriate diagnostic tools are lacking.

Macrophages are crucial to the pathogenesis of mycobacteria since they serve as a perfect ecosystem for Mtb replication. Alveolar macrophages are the primary responders that internalize the aerosolized Mtb and utilize multiple mechanisms such as the synthesis of cytokines/chemokines, reactive oxygen and nitrogen species, phagosome lysosome fusion and autophagy to eliminate the bacteria. However, these cells are not efficient in eradicating the bacteria since the bacteria have evolved strategies to breach the bactericidal effects of the macrophages. The bacteria continue to multiply intracellularly and ultimately cause macrophage disruption, thereby infecting the nearby cells. Macrophages, DCs, fibroblasts and other immune cells accumulate at the site of infection and aggregate to form the granulomatous structure (16, 20).

**Figure 2:**
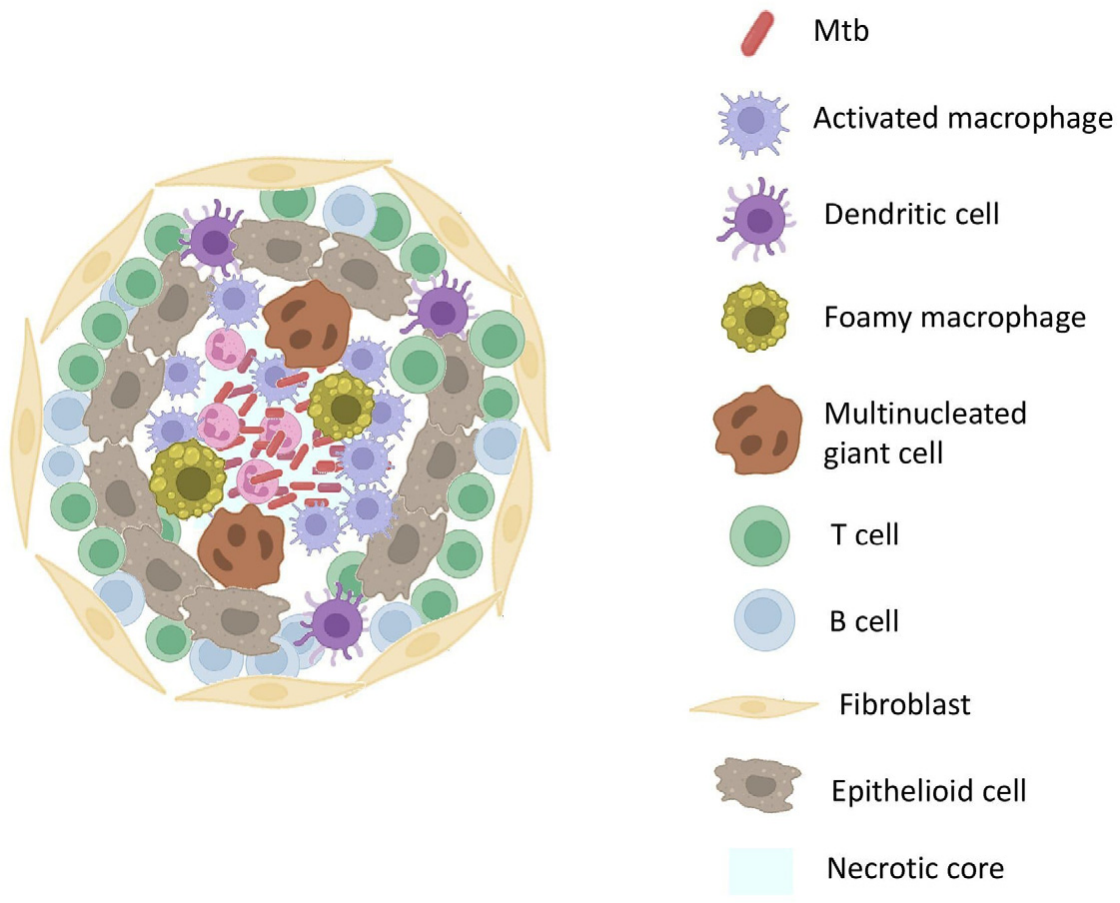
Structure Of TB GranulomaThe core of the granuloma contains necrotic tissue surrounded by activated macrophages. Surrounding the core are layers of epithelioid macrophages and multinucleated giant cells, encapsulated by a layer of lymphocytes. The outermost layer comprises fibrotic tissue, providing structural stability.

In the granuloma illustrated in **Figure 2**, macrophages differentiate into heterogeneous phenotypes such as epithelioid cells, foamy macrophages and/or fuse to form multinucleated giant cells (MGCs). The lipid-rich foamy macrophages were found to be associated with necrotic lesions and provide a nutrient-rich reservoir for Mtb. This strongly highlights the role of lipid accumulation in TB pathogenesis (21). MGCs form the central core of the granuloma. Interestingly, these cells were absent in disseminated TB suggesting their role as a niche for LTBI (22). These cells in turn elicit an inflammatory response and release a myriad of chemoattractants that attract other innate cells like neutrophils and DCs. DCs, the most efficient T-cell stimulators, capture the antigens and reach the local lymph nodes to prime the naïve CD4+ T-cells. DCs constitute the primary link between innate and adaptive immunity. They successfully accomplish this by not only providing antigen-specific stimulation but also by providing secondary and tertiary signals to sensitize T-cells (23).

Activated T and B cells reach the granuloma and surround the innate immune cells contributing to its outermost layer (24). However, in an interesting twist, the bacteria turns the host protective structure, granuloma into a self-protective shell where it finds nutrients to facilitate its own growth and transmission (25). This is due to the lag in the initiation of adaptive immune response. As is typical for other infections, the adaptive immune response to Mtb is triggered in the draining lymph nodes rather than the developing lung granuloma and this attributed to the delayed onset of primed T cells to the lungs (26).

Among the innate cells, neutrophils play a negative role and its activity was found to have a robust impact on TB infection outcomes with recent findings highlighting its significance in disease progression. Examining the gene expression profiles of patients with latent and active TB highlights a unique neutrophil-driven signature, which is especially evident in those with active disease marked by an increase in the interferon-beta (IFN-β) (20, 27). In mice models infected with Mtb, early depletion of neutrophils led to increased frequency of Mtb-laden DCs in the lung whereas decreased migration of DCs to lymph nodes thus further delaying the priming of T-cells (28).

### 4.2 Adaptive immune response against Mtb

Antigen presentation to T-cells activates the adaptive immune response. As discussed above, cell-mediated immunity performs a dual role by providing protective immunity to the host and contributing to the development of caseous necrotic lesions required for Mtb pathogenesis. Adaptive cell-mediated immunity comprises mainly of CD4+ and CD8+ cells, with antigen-specific CD4+ cells which are the major producers of IFN-γ playing an important role in anti-tuberculous immunity. Despite CD4+ cells, CD8+ and NK cells also contribute to IFN-γ production (29). IFN-γ exerts its protection through macrophage activation. Currently, quantifying the IFN-γ released by these cells in response to Mtb-specific antigens is used as a gold standard test to detect LTBI. IGRA positivity rates will provide valuable data on the burden of LTBI within a population (30).

CD8+ cells, like CD4+ cells, are capable of releasing potent macrophage-activating cytokines that aid in the control and elimination of intracellular pathogens like Mtb (31). These cells also employ direct killing mechanisms by releasing cytolytic proteins such as granulysin. CD8+ cells typically utilize cytolytic pathway over cytokine production and preferentially recognize heavily infected cells whereas CD4+ cells prioritize cytokine production. Because they prefer cytotoxic activity, CD8+ cells act as important mediators of immune surveillance by actively recognizing and inducing apoptosis in infected cells depriving Mtb of a conducive environment (32). As Mtb transitions from latency to active state, the number of highly infected cells in the host probably increases. Since the immune surveillant CD8+ T cells prefer heavily infected cells, they might become more frequent in number in response to this rise in infected cells (31).

In a remarkable observation, CD4+ cells exhibited heightened activity during the acute phase of the infection. On the other hand, CD8+ T cells play a less significant role in the acute stage and become activated and start producing IFN- γ during the latent phase of infection. This points to a change in the immune response dynamics with CD8+ T cells becoming increasingly involved during the latent phase of Mtb infection, and CD4+ T cells being primarily active during the acute phase. Studies like the Cornell model of TB demonstrate the reactivation of LTBI following the depletion of CD8+ T cells proving the crucial role of CD8+ T cells in maintaining latency and preventing disease progression (16, 33, 34). The protection the CD4+ T cell fraction provides is significantly higher than that of CD8+ T cells in various experimental settings. A study employing adoptive transfer of CD8+ enhanced cells derived from mice infected with Mtb has indicated that this subset can contribute to only a modest reduction in the bacterial load (35).

The humoral adaptive immunity consists of B cells which mediate its protection through antibody production, antigen presentation, cytokine production and modulating T cell responses (36). Activated B cells are found in the granuloma of Mtb-infected murine host models (37). They play a role in immunomodulation via the production of cytokines like IL-10 and activation of FcγR to enhance the effector function of T cells (38). The classic granuloma is a caseous necrotic lesion with a hypoxic region in the center. In the latent state, bacteria were found to be residing in the center hypoxic region in a dormant state whereas active TB was characterized by bacteria multiplying along the margins of liquid cavities (11). Both innate and adaptive immunity take part in the formation of granuloma and their role in protection and pathogenesis is not very well described.

## 5 Inflammatory response in Mtb infection

Infection by Mtb causes disruptions in the homeostasis of host tissue, which activates immune surveillance systems that fuel inflammation. Different clinical manifestations of TB disease trigger the immune response leading to systemic inflammation. Certain autoantibodies have been found to be increased in TB patients, implying that the infection and autoimmune responses are linked. The mechanisms underlying this could include immune system dysregulation in response to the prolonged infection, which can contribute to tissue damage and inflammation. This inflammation is characterized by the release of various inflammatory mediators such as acute phase proteins (APPs), lipid mediators and a range of pro- and anti-inflammatory cytokines and chemokines into the bloodstream. This notable increase in soluble mediators attracts various immune cells to the afflicted tissues which is often reflected by a strong inflammatory signature that can be observed through the analysis of inflammatory markers and cell populations in peripheral blood samples (39, 40). Inflammation is an important factor determining the outcome of Mtb infection. Excessive inflammation can cause tissue damage, leading to complications like lung cavitation. On the other hand, insufficient or lack of inflammation could worsen the infection by allowing the bacteria to replicate unchecked (18).

### 5.1 Role of cytokines

Cytokines and chemokines play a vital role in regulating TB by aiding in efficient cell migration and providing specific guidance during the immune response. The functions and interactions between these components are essential for curbing TB, whereas large bacterial burdens could exploit this host cytokine signaling for proliferation and invasion hindering efficient cell coordination and immune reaction (41). Cytokine production is initiated by various immune cell interaction with Mtb. Within these cells, multiple pattern-recognition receptors (PRRs) simultaneously sense and recognize a range of Mtb-encoded factors promoting the production of specific cytokine profile (42). Cytokines are broadly classified into two types based on their roles in inflammation: pro-inflammatory and anti-inflammatory. Pro-inflammatory cytokines act as alarm signals, initiating the immune response by activating other immune cells to contain and eliminate bacteria. In contrast, anti-inflammatory cytokines function as regulators, preventing excessive tissue damage and inflammation during the immune response. Maintaining a balance between pro-inflammatory and anti-inflammatory cytokines is vital for effectively combating Mtb while preventing excessive tissue damage. An excessive pro-inflammatory response can lead to immunopathology, causing damage to host tissues. Conversely, an overly anti-inflammatory response may enable immune evasion, allowing Mtb to escape immune surveillance and persist despite the adaptive immune response (43).

Among the pro-inflammatory cytokines, IFN-*γ* and TNF-*α* are recognized as key players in the antimycobacterial cytokine cascade. TNF-α works synergistically with IFN-γ to facilitate the formation of granulomas, which are essential for containing and controlling mycobacterial infections (44). These cytokines activate specific signaling pathways which shapes the host response to Mtb. The most important pathways being STAT1 pathway involved in IFN-*γ* signaling and NF-κB Pathway in TNF-α signaling. IFN-γ binds to its receptor, leading to the activation of the JAK-STAT pathway. Specifically, STAT1 is phosphorylated, dimerized and translocated to the nucleus to induce the expression of genes critical for antimicrobial activity. Blocking IFN-γ mediated signaling is an important immune evasion strategy employed by Mtb. This can dampen the host’s ability to control bacterial replication and promote survival within macrophages. TNF-α binds to its receptor, leading to the activation of the NF-κB signaling cascade. This results in the transcription of pro-inflammatory genes that recruit immune cells to the site of infection. Mtb may exploit this balance, modulating the pathway to ensure its persistence (45). Anti-inflammatory cytokines, particularly IL-10 and TGF- β regulates inflammation, cell proliferation and migration. TGFβ can also induce IL-10 and synergize with it to suppress IFNγ production, suggesting that it plays both a regulatory role and potentially a negative role in the context of Mtb infection (41). Cytokines and their various roles in TB disease has been tabulated in **Table 2**.

**Table 2:** List of cytokines, cellular sources and their role in the pathophysiology of TB.

CYTOKINE	SOURCE	ROLE IN TB
Type 1 IFN (IFN-α/β)	Innate immune cells	Reduced expression - Host protection by decreasing Th1 immunity, Increased expression - Disease pathogenesis and bacterial expansion (46)
Type II IFN (IFN-γ)	Th1 cells, NK cells, pulmonary epithelial cells	Pro-inflammatory, Bactericidal action, macrophage activation and induction of phagocytosis (47)
IL-2	Th1 cells	Protective action against Mtb infection (48)
TNF-α	Mononuclear phagocytes, Alveolar Epithelial cells (AECs), Alveolar macrophages, DC, T cells	Pro-inflammatory, phagosome maturation, early granuloma formation, decreased production - fatal TB outcome (49)
IL-1 (IL-1α/IL-1β)	Monocytes, macrophages, neutrophils, endothelial cells, DC, alveolar macrophages	Pro-inflammatory, early IL-1 responses might be important for protection, at a later stage, they contribute to tissue damage (50)
IL-6	Endothelial cells, lymphoid and non-lymphoid cells, fibroblasts	Pro-inflammatory, early protective responses, Th17 differentiation, acute phase response (51, 52)
IL-12	DC, monocytes	Pro-inflammatory, enhances IFN-γ production, Th1 immune response (53)
IL-17A	T cells primarily γ/δ T cells, NK cells	Pro-inflammatory, formation of mature granuloma, neutrophil recruitment via maintenance of chemokine gradients (54, 55)
GM-CSF	T cells, macrophages, AECs	Granuloma formation, Monocyte/Macrophage proliferation, induction of autophagy (56)
IL-18	Macrophages, DC	Pro-inflammatory, contributes to optimal IFN-γ secretion, NK cell activation, Th1 response (42, 57)
IL-4	Th2 cells	Macrophage activation (58)
IL-10	Th2 cells, neutrophils	Anti-inflammatory, inhibits IFN-γ secretion and T cell proliferation, downregulates immune response to TB (59)
TGF- β	Monocytes, T reg cells	Anti-inflammatory, macrophage deactivation, T cell suppression (60)
IL-22	NK cells, macrophages, γδ T cells, Th1, Th17 and Th22 cells	Pro-inflammatory, enhances TNF-α production, mycobacterial growth inhibition (61)
IL-23	DC	Enhances IL-17 production, induces antimycobacterial IFN-γ (62, 63)
IL-27	DC, macrophages	Both pro- and anti-inflammatory, negative regulator of autophagy, suppresses TNF-α and IL-12 production (64)
IL-32	T cells, NK cells	Pro-inflammatory, control of Mtb infection, reduces tissue damage (65)
IL-35	Treg cells, DC, B cells, macrophages	Anti-inflammatory, immunosuppression (66)

### 5.2 Role of chemokines

Chemokines are chemoattractant molecules that serve as signaling agents guiding the migration of immune cells to the site of infection. They act in a concentration gradient where cells move toward the chemokine’s higher concentration. However, cell migration and chemokine concentration do not have a linear connection. Cells can only respond up to a certain threshold of chemokine concentration. Desensitization of receptors happens in extreme cases reducing the cell’s ability to migrate in response to subsequent signals (67). Homeostatic/constitutive chemokines, as the name suggests, play an integral role in maintaining tissue integrity and homeostasis. In contrast to homeostatic chemokines, inflammatory chemokines are inducible and only operate at the site of inflammation by recruiting leucocytes. Their expression is tightly regulated and is essential for mounting effective immune responses following an inflammatory insult (68). Different chemokines play distinct functions in attracting various types of immune cells to ensure a coordinated and targeted response to infections or tissue injury. Mtb is a potent inducer of chemokine expression. CCL1-5 and CXCL1-11 belong to the inflammatory chemokine subfamily Typically, CXCL1-8 governs the migration of neutrophils whereas CXCL9-11 directs the migration of activated T cells (69).

Chemokines and their receptors orchestrate the recruitment of immune cells to infection sites. For instance, CCL2 attracts monocytes, while CXCL10 recruits T cells. However, Mtb manipulates these pathways by inducing CXCR3 and CCR2 desensitization, impairing effective immune cell trafficking. This disruption undermines granuloma formation and facilitates bacterial persistence. Furthermore, Mtb can distort chemokine gradients to misdirect immune cells, reducing the efficiency of the host response (70). A summary of the chemokines and their receptors are given in **Table 3**.

**Table 3:** Summary of chemokines and their function in TB.

CHEMOKINE	SOURCE	RECEPTOR	CELLULAR EXPRESSION	ROLE IN TB
CCL1	Activated T cells	CCR8	Th2 cells and innate lymphoid cells (ILCs)	Monocyte chemotaxis (71)
CCL2	Monocytes, macrophages and DC	CCR2	Monocytes, macrophages, CD4+ T cells and immature DC	Granuloma formation, Leucocyte activation (72)
CCL3	Monocytes, macrophages and DC	CCR1/CCR5	Macrophages, lymphocytes, and eosinophils	Th1 cell differentiation (73, 74)
CCL4	T cells, monocytes and lymphocytes	CCR5	Th1 cells, monocytes and lymphocytes	Th2 cell migration (75)
CCL5	T cells, monocytes	CCR1/CCR3/CCR4/CCR5	Macrophages, monocytes, CD4 T cells, CD8 T cells	Early protective Mtb*-*specific immunity, granuloma formation (76, 77)
CXCL1	Macrophage, neutrophils and Th17 cells	CXCR1/ CXCR2	Neutrophils	Neutrophil chemoattractant (78, 79)
CXCL2	Macrophage, mast cells, Type II AECs	CXCR2	Neutrophils	Neutrophil chemoattractant (79)
CXCL3	Macrophage, DC	CXCR2	Neutrophils	Neutrophil chemoattractant (80)
CXCL5	Type I and type II AECs, macrophages	CXCR2	Neutrophils	Neutrophil chemoattractant (81)
CXCL6		CXCR1/ CXCR2	Neutrophils	Neutrophil chemoattractant (82)
CXCL7	Monocytes, macrophage, neutrophils, NK cells	CXCR2	Neutrophils	Neutrophil chemoattractant (82, 83)
CXCL8	Monocytes, pulmonary fibroblasts	CXCR1/ CXCR2	Neutrophils	Granuloma formation, monocyte, lymphocyte, and neutrophil chemoattractant (84)
CXCL9	Monocytes, fibroblasts	CXCR3	Monocytes, Th1 cells, CD4 T cells, CD8 T cells, NK cells, DC	Granuloma formation, early marker of IFN-γ activation (85–87)
CXCL10	Monocytes, lymphocytes, fibroblasts	CXCR3	Monocytes, Th1 cells, CD4 T cells, CD8 T cells, NK cells, DC	Granuloma formation, T cell migration and NK cell stimulation (86, 88, 89)
CXCL11	Monocytes, fibroblasts	CXCR3	Monocytes, Th1 cells, CD4 T cells, CD8 T cells, NK cells, DC	NK cell stimulation and T cell chemoattractant (89)

## 6 Cytokine and chemokine responses during latent and active TB

The immunological mechanisms underlying the development of latent or active TB are complex and not fully understood. Several recent studies have elucidated the changes in the cytokine and chemokine profiles during latency and active states of TB. Some of the findings from these studies are briefly reported here. Peripheral blood mononuclear cells (PBMCs) produced a distinct Type I cytokine profile in response to Mtb-specific antigens and stimulation with Mtb soluble extract (MTSE) increased the percentage of IFN-γ/IL-17 producing NK cells and ILCs in active TB. These profiles were not found in healthy volunteers or LTB patients, suggesting that they could help differentiate between latent and active TB (90). However, in a study by Marin et al., they found an increase in the frequency of IFN-γ producing Th1 cells in LTBI, whereas IL-17 producing Th17 cells were more common in TB patients suggesting how Mtb alters the host profile towards the pathological Th17 response rather than the protective Th1 profile (91) In our previous study, we observed a progressive increase in IL-17 levels across the TB disease spectrum, ranging from latent infection to drug-resistant TB (DR-TB). IL-17 exhibited AUC of 0.97 and significantly discriminate DR-TB from drug-sensitive TB (DS-TB). Additionally, we demonstrated significant changes in plasma cytokine levels during the transition from latency to DS-TB or DR-TB. Notably, we identified a unique plasma cytokine signature - comprising IL-17, IL-1α, IL-2, IL-10, IL-5, IFN-γ, TNF-α, and IL-6 - that is specific to distinct stages of TB disease. Remarkably, these cytokines exhibited high diagnostic accuracy, with AUC values exceeding 0.8, highlighting their potential as reliable biomarkers (92).

**Figure 3:**
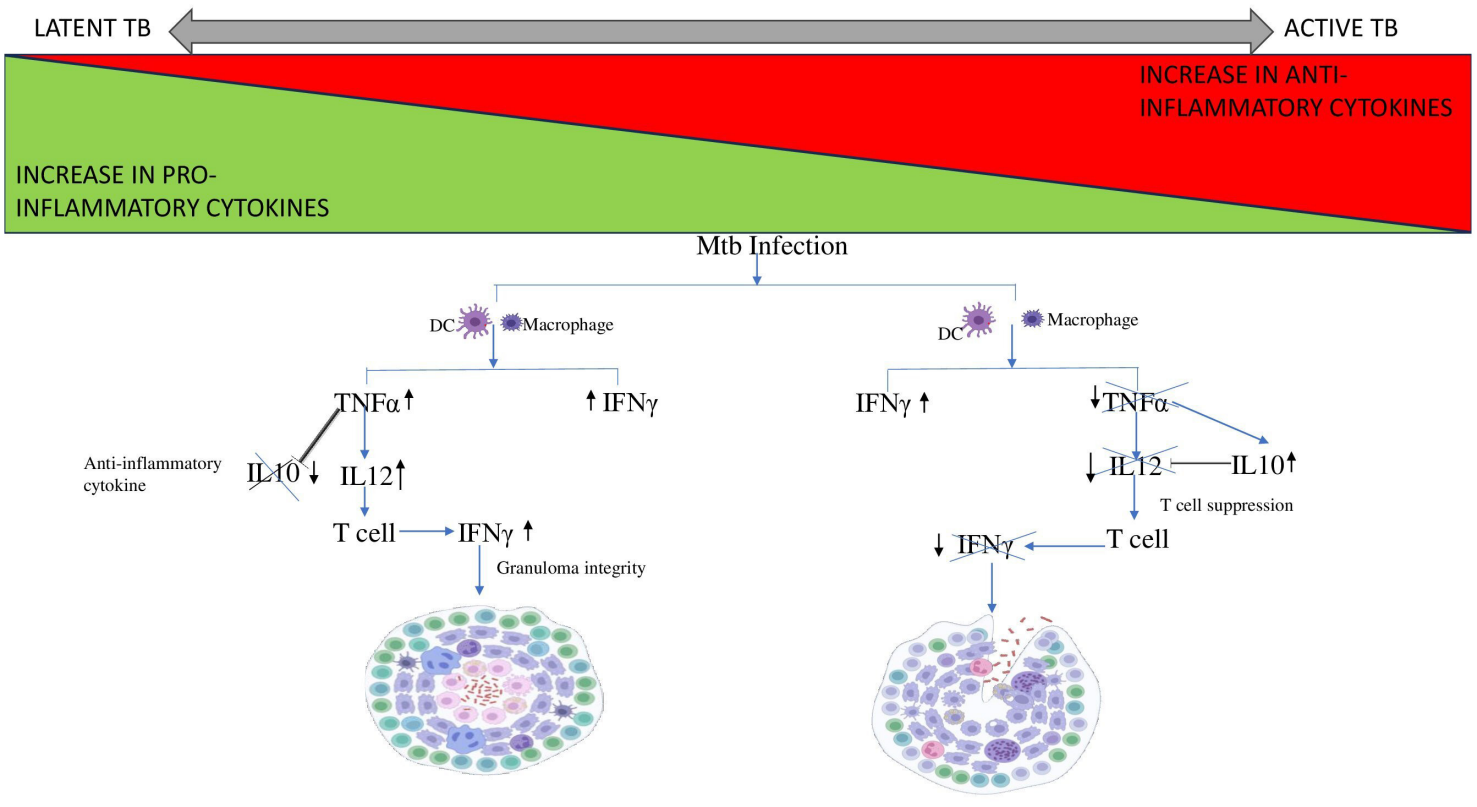
Cytokine cascade in Latent and Active TB. Mtb infection leads to the recruitment of DC and macrophages to the site of infection. In case of LTB, the increase in pro-inflammatory cytokine TNF-α stimulates IL12 expression and supresses the expression of anti-inflammatory cytokine, IL10 thus preventing it from interfering with granuloma integrity. Whereas, in case of active TB, TNF-α levels decreases thus is unable to supress IL10. Therefore, IL10 levels increase and inhibits IL12 leading to T cell suppression. Thus, the T cell fails to produce IFN-γ resulting in disintegration of the granuloma.

The balance between proinflammatory and anti-inflammatory cytokines secreted by phagocytes after exposure to Mtb antigens dictates T cell activation and granuloma formation (**Figure 3**) (53). TNF-α influences immune cell trafficking and promotes the formation of organized granulomatous structure efficient in disease control and helps maintain the granuloma integrity in LTBI. TNF-α mediated granuloma formation is regulated by chemokines CCL2, CCL3, CCL4, CCL5 (CC chemokines) and CXCL8, CXCL9, CXCL10 (CXC chemokines) which recruits leucocytes to the site of granuloma (93). Elevated CXCL9 levels are often observed in diseased groups and thus help determine the disease severity (93, 94). The Cornell model of LTB provided good evidence for the role of TNF-α in controlling LTBI (34). In another example, the neutralization of TNF-α led to the fatal reactivation of persistent LTBI, triggering the increased expression of anti-inflammatory cytokine IL-10, which is typically associated with severe pathology (95). IL-12 regulates the production of IFN-γ and the cytotoxic effector function of T cells. IL-12 production is optimal in early infection, but down-modulated in monocytes exposed to increased levels of IL-10 and TGF-β thus leading to immunologic unresponsiveness and failure to produce IFN-γ (96, 97). Neutralization of IL-10 enhances the production of IFN-γ by increasing IL-12 production by monocytes, which then activates T cells to further increase IFN-γ production (97). In contrast to TNF-α and IFN-γ, TGF-β promotes the growth of Mtb intracellularly by suppressing T cell responses and causing macrophage deactivation. An earlier study reported the constitutive expression of TGF-β in monocytes from patients with active TB (60, 98). Anti-inflammatory cytokines, TGF-β1 and IL-10 have been shown to regulate lymphoid-derived cells and myeloid-derived cells respectively in other diseases which could help us better understand mycobacterial persistence in TB (99, 100).

T cells are polyfunctional and can produce more than one cytokine sequentially in response to infection/stimulation. A decline in activated macrophages and a concurrent rise in T cells may be indicative of advanced and severe forms of cavitary TB disease. Lung tissue from patients with latent tuberculous granulomas was found to be less macrophagic and more enriched with lymphocytes (101).

An expansion of CD4+IFN-γ+IL-17+ lymphocytes during active TB is correlated with the disease severity (102). In humans, CD4+ T cells secreting IL-17 selectively express CCR6 and CCR4 whereas CCR6 and CXCR3 are expressed in Th1 cells producing IFN-γ and T cells producing both IFN-γ and IL-17. In the case of LTBI, memory CD4+ T cells are strongly biased towards the Th1 subset expressing both CCR6 and CXCR3 (103). In addition to CD4+ cells, CD8+ cells were also observed to be at increased frequency upon Mtb infection. The cytokines, IFN-γ, IL-2, and TNF-α were generated simultaneously by the majority of CD8+ T cells. While the chemokine receptors CCR4, CXCR3, and CXCR4 might not be involved in the selective migration of CD8+ T cells, CCR6 may help these cells migrate into localized TB-infected sites (104). In murine models, depletion of CD8+ cells significantly reduced their ability to control recurrent infection whereas adoptive transfer of CD8+ T cells confers protection against Mtb (105).

Despite these results, currently, there are no biomarkers available to accurately distinguish the different phases of TB infection. Among the wide range of biomarkers, IP-10 is one of the most extensively studied alternative biomarkers to improve the diagnostic performance of IGRA. Its expression is reportedly 100 times higher than IFN-γ making it highly sensitive for detection. The development of two whole-blood IGRAs by R-Biopharm, utilizing either ELISA or lateral flow assay, represents a practical application of IP-10 as a biomarker (106, 107). Details from a systematic review of the Mtb-specific cytokine biomarkers revealed few of the significant combinations of cytokines IL-2 and IFN-γ, IL10 and IFN-γ, IFN-γ and IP-10, IFN-γ and TNF-α, and IL-2 and TNF-α which shows potential in discriminating between active TB and LTBI (108). One study found a combination of biomarkers (IL-5, IL-10, TNF-a, VEGF, and IL-2/IFN-γ) that demonstrates strong predictive capability for distinguishing between latent and active TB. The high accuracy percentages - 93.3% for latent TB and 95.5% for active TB - highlight the potential for these markers to contribute to improved diagnostic strategies. Particularly, VEGF showed great ability to differentiate active TB regardless of stimulation suggesting it could be a key standalone indicator in future diagnostic models (109). There are several ongoing trials of serum biomarkers that have the potential to revolutionize TB diagnostics. However, extensive validation studies in diverse settings are necessary to ensure global applicability.

## 7 Role of APPs

**Figure 4:**
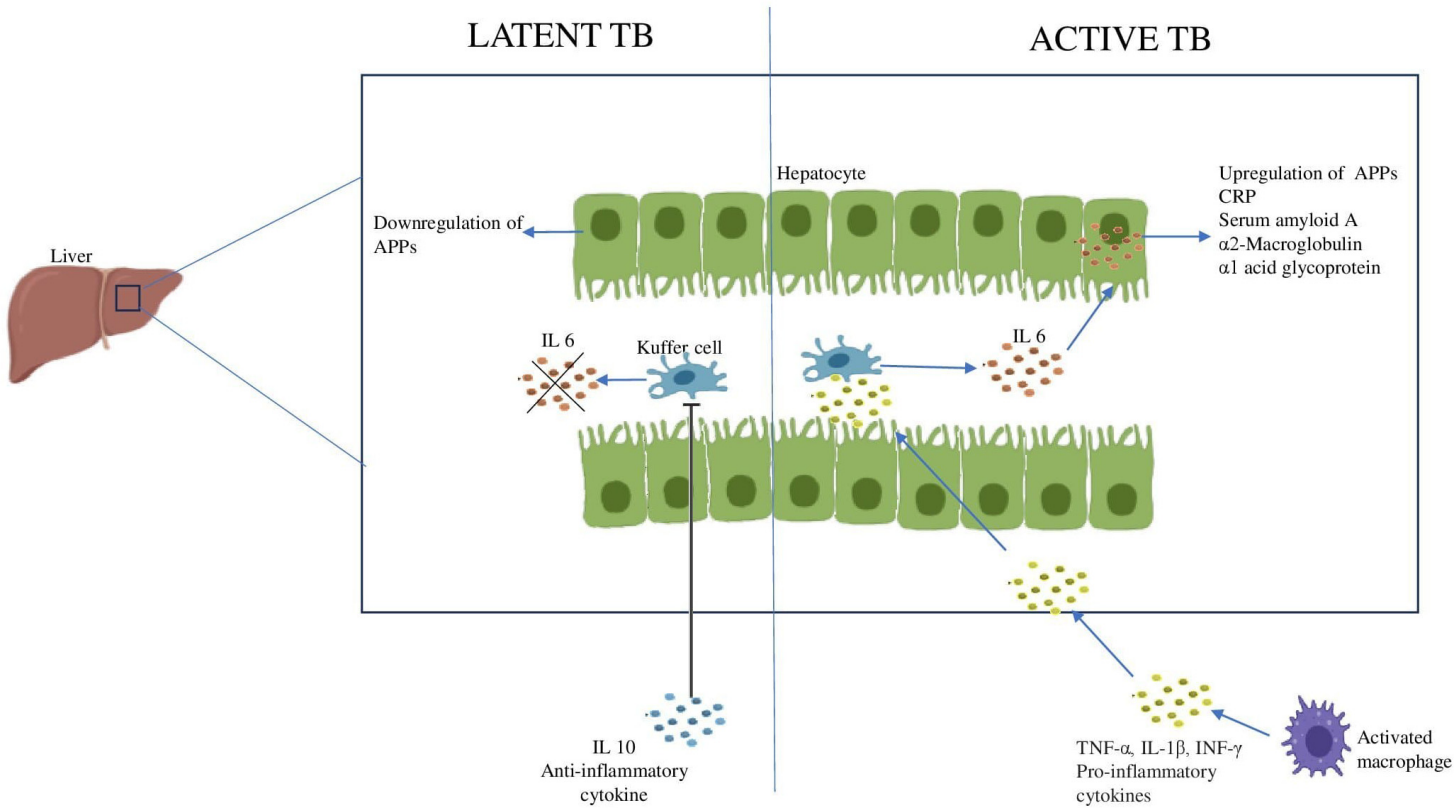
Acute phase response during Latent and Active TB IL6 produced by the Kuffer cells of the liver stimulates the hepatocytes to produce APPs. The increase in anti-inflammatory cytokine, IL10 inhibits the Kuffer cells leading to downregulation of APPs.

The immunological hallmark of TB is the release of inflammatory mediators that stimulate the acute phase response (APR) (110). Generally, the liver produces APPs in response to stress as part of the body’s inflammatory response and their systemic levels reflect the levels of pro-inflammatory cytokines in the body (111). The inflammatory cytokines produced during TB disease stimulate the Kuffer cells of the liver to secrete IL-6. Therefore, IL-6 is the primary cytokine responsible for the majority of APP hepatocytic production. Resolution of APR is achieved by the anti-inflammatory cytokines like IL-10 which regulate the negative response (112, 113). However, it has been observed that during LTBI, IL-6 levels are reduced and IL-10 levels are increased, which may account for the lower expression of APPs in those who are latently infected (**Figure 4**) (114).

The liver produces a large number of APPs while simultaneously reducing the production of other proteins. Depending on their serum concentration during inflammation, they are categorized as either positive APP or negative APP which are upregulated or downregulated respectively (115). In a study conducted among 126 individuals, elevated levels of procalcitonin, CRP and α1 acid glycoprotein have been reported to distinguish between active TB and LTBI (116). A systematic review and meta-analysis of APPs and cytokines found CRP to be an efficient candidate for the diagnosis of TB. CRP is already used in clinical practice as a diagnostic marker for pediatric TB (117). Two additional APPs, α-2-macroglobulin and haptoglobin, are also differentially expressed and could complement CRP in the evaluation of TB (118). Another study conducted among the urban Tanzanian LTBI population revealed a higher concentration of α1 acid glycoprotein suggesting its use as a marker of inflammation in LTBI (119). This finding needs to be evaluated whether individuals with LTBI and elevated α1 acid glycoprotein are in the early stages of active TB or if LTBI itself could be contributing to chronic low-grade inflammation. The role of the major APPs in TB has been summarized in **Table 4**.

**Table 4:** Acute phase proteins and their regulatory mechanisms in controlling TB.

APP	ROLE IN TB
POSITIVE	
C-reactive protein (CRP)	Marker of bacterial inflammation, associated with large Mtb burden and increased frequency of disseminated TB (115, 120)
Procalcitonin	Increased secretion during bacterial infection and decreased during viral infection (121)
Fibrinogen	Associated with a large Mtb burden, tissue damage and degree of inflammation (122)
α1 acid glycoprotein	Granuloma formation and influences T cell function (123)
Serum amyloid A (SAA)	Associated with disease severity, promoting amyloid deposition in granuloma (124)
Haptoglobin	Immunoregulatory effect, suppresses lymphocyte proliferation, together with Transferrin detains iron availability to invading bacteria (125)
α2-Macroglobulin	Marker of disease progression in malnourished individuals (126)
Mannose-binding protein (MBP)	Low levels offer protection and elevated levels aid the survival of Mtb within macrophages (127)
NEGATIVE	
Albumin	Together with transferrin, decreases zinc and iron availability to bacteria (128)
Retinol-binding protein (RBP)	Retinol transporter (129)
Apolipoprotein A	Marker of TB infection (130)
Transferrin	Sequesters Fe to inhibit Mtb growth (128)

## 8 Inflammation - boon or bane?

The pathophysiology of TB is thought to be influenced by a disruption of the delicate balance between inflammatory response and host defense. Both elevated and lowered inflammatory responses may increase the host’s vulnerability to the infection (131). Uncontrolled inflammation not only compromises host immunity but also disrupts cellular and systemic metabolic equilibrium and the ability of the host to eradicate infection. Th1 response typically helps contain the disease by activating the macrophages. Nevertheless, in some cases, an overly robust Th1 response may not be able to eradicate the bacteria and may even worsen the illness and cause tissue damage (132). The granuloma triggered by Mtb infection can have both protective and harmful consequences. From a protective perspective, it involves the infiltration of inflammatory cells to the primary site of infection to form granuloma. This structure encapsulates and localizes the bacteria, preventing dissemination to other parts of the body. On the other hand, the granuloma provides a safe niche for the intracellular survival of bacteria. Once the immune system weakens, disruption of protective granuloma can occur, facilitating bacterial dissemination and progression to active TB which leads to an adverse inflammatory response and extensive tissue damage. This imbalance between protective and destructive host responses causes variability in clinical presentation and a higher incidence of LTBI (133).

Nearly all components of the immune system participate in the fight against Mtb. They utilize several inflammatory mechanisms to protect the host. Unfortunately, the same is exploited by Mtb to be used as a destructive tool to progress inside the host. It remains a debate whether the inflammation generated during TB is protective for the host or detrimental. This demonstrates the complexities of TB pathogenesis and the challenges in creating potent treatments and vaccinations to combat this enduring infection.

## 9 Research gaps and future directions

Despite having an increasing knowledge of the role of inflammation during disease and the molecular mechanisms behind inflammatory processes, several questions remain unanswered. 1) What is the role of inflammation in the persistence of LTBI and its sudden reactivation? 2) How does granuloma contribute to protection or pathogenesis? 3) How does inflammation increase or decrease susceptibility to infection? 4) Since these inflammatory markers play a vital role throughout the disease state, whether these can be utilized as biomarkers for diagnosis and for discriminating between different substates of TB? and 5) Whether the anti-inflammatory drugs can be used as an adjunct host-directed therapy for TB? Future research should aim at conducting longitudinal cohort studies that follow people with LTBI over time to better understand the factors that predict progression to active illness. The observed biomarkers must be validated in a larger cohort and across different ethnic groups to ensure their applicability and reliability. The application of genomics and other “omics” technologies to find novel genetic or transcriptome signs of LTBI could lead to the creation of more sensitive diagnostic assays. This could be combined with the immunological and environmental data to determine who is at risk.

A key challenge in host-pathogen interaction is the morphological variability of differentially polarized primary human monocyte-derived macrophages (hMDMs). In such cases, spatio-temporal analysis offers a sophisticated method to study the interaction between Mtb and human macrophages by utilizing micropatterning to standardize macrophage morphology and organelle positioning. This technique minimizes variability and enables high-resolution, high-throughput analysis (134). Di Zhang et al. showed how spatially mapping multiple layers of omics information over different time points provides critical insights into the inflammatory processes shaping neural architecture and function in the brain (135). However, these are beyond the scope of this review. In the past decades, though several inflammatory biomarkers were investigated for their potential to diagnose TB and to discriminate between active TB and LTBI, they were found to be inferior to the direct detection of Mtb. Some promising candidates have been mentioned but several inconsistencies are reported among different research findings making them unsuitable for technical application. More stringent investigations are required in the quest for biomarkers for the rapid detection of Mtb and monitoring treatment efficacy.

## 10 Conclusion

In the case of LTBI, inflammation is the one that decides the ultimate fate of the host and of the pathogen. Thus, rather than targeting the pathogen, inflammatory imbalances need to be addressed in order to successfully treat the condition without damaging the host. Both hypo- and hyper-inflammation are harmful to the host and interventions focusing on balancing inflammation should be the target for future host-directed therapies to efficiently control the infection.

While this review provides a thorough examination of the circulating markers, certain limitations must be acknowledged. These circulating inflammatory markers are also implicated in other inflammatory conditions like Rheumatoid arthritis and other diseases, their varying expression levels may not be exclusive to TB, thereby affecting the specificity percentage. In addition, the variability could also stem from various other confounding factors such as sample size, technical disparities, ethnicity, individual inflammatory levels, as well as dietary and lifestyle patterns. And also, the scarcity of longitudinal studies limited our ability to assess long-term outcomes. We made a concerted effort to identify superior candidates studied across various stages of TB disease. However, these candidates require further validation across multiple cohorts using a multi-centric approach to ensure their suitability for clinical application.
